# TNF*α* Mediates the Interaction of Telomeres and Mitochondria Induced by Hyperglycemia: A Rural Community-Based Cross-Sectional Study

**DOI:** 10.1155/2020/8235873

**Published:** 2020-05-04

**Authors:** Lu Lyu, Shuli He, Huabing Zhang, Wei Li, Jingbo Zeng, Fan Ping, Yu-Xiu Li

**Affiliations:** ^1^Key Laboratory of Endocrinology, Ministry of Health, Department of Endocrinology, Peking Union Medical College Hospital, Peking Union Medical College, Chinese Academy of Medical Sciences, Beijing 100730, China; ^2^Department of Clinical Nutrition, Peking Union Medical College Hospital, Peking Union Medical College, Chinese Academy of Medical Sciences, Beijing 100730, China; ^3^Department of Endocrinology, Fuxing Hospital, The Eighth Clinical Medical College, Capital Medical University, Beijing 100038, China

## Abstract

This study is aimed at evaluating the relationship between leukocyte telomere length (LTL) and mitochondrial DNA copy number (mtDNAcn) in a noninterventional rural community of China with different glucose tolerance statuses. In addition, we investigate whether the indicators of oxidative stress and inflammation were involved and identify mediators among them. A total of 450 subjects in rural China were included and divided into two groups according to a 75 g oral glucose tolerance test (OGTT): the abnormal glucose metabolism (AGM, *n* = 257, 57.1%) group and the normal glucose tolerance (NGT, *n* = 193, 42.9%) group. Indicators of oxidative stress (superoxide dismutase (SOD) and glutathione reductase (GR)) and inflammatory indices (tumor necrosis factor *α* (TNF*α*) and interleukin-6 (IL-6)) were all determined by ELISA. LTL and mtDNAcn were measured using a real-time PCR assay. Linear regressions were used to adjust for covariates that might affect the relationship between LTL and mtDNAcn. Mediation analyses were utilized to evaluate the mediators. In the AGM, LTL was correlated with mtDNAcn (*r* = 0.214, *p* = 0.001), but no correlation was found in the NGT. The association between LTL and mtDNAcn was weakened after adjusting for inflammatory factors in the AGM (*p* = 0.087). LTL and mtDNAcn were both inversely related to HbA1c, IL-6, TNF*α*, and SOD activity. Mediation analysis demonstrated that TNF*α* was a significant mediator in the telomere-mitochondrial interactome in the AGM. This result suggests that inflammation and oxidative stress may play a vital role in telomere shortening as well as mitochondrial dysfunction. In the subjects with hyperglycemia, a significant positive correlation is observed between LTL and mtDNAcn, which is probably mediated by TNF*α*. TNF*α* may be considered a potential therapeutic target against aging-related disease in hyperglycemia.

## 1. Introduction

Type 2 Diabetes (T2DM) is a worldwide epidemic characterized by insulin resistance and abnormal insulin secretion, which can result in severe complications and increased medical care costs. Unfortunately, China has become the world's most massive diabetes epidemic since the prevalence of T2DM increased at a substantial rate, which was primarily driven by population aging. Despite the fact that diabetes was more common in urban areas, it was the rural areas that were associated with higher diabetes-related mortality [1].

Telomere damage and mitochondrial dysfunction are both hallmarks of aging. In the past ten years, these two hallmarks were studied, respectively. Recently, a few reports have revealed that there are profound links between telomere attrition and mitochondrial reprogramming, which promote their interaction in aging and degenerative diseases [2]. Previous studies suggest that certain cytokines shuttle between the nucleus and mitochondria upon oxidative stress, which may influence both telomere biology and mitochondrial function [3]. Meanwhile, oxidative stress and inflammatory responses were both involved in the onset and progression of T2DM. However, the specific factors involved in oxidative stress or inflammation contributing to the malfunction of the mitochondrial-telomere axis remain unclear.

The present study sought to assess the relationship between leukocyte telomere length (LTL) and mitochondrial DNA copy number (mtDNAcn) based on a noninterventional rural population with different oral glucose tolerance statuses. The indicators of oxidative stress or inflammatory cytokines involved in the interaction of telomere attrition and mitochondrial dysfunction were also analysed.

## 2. Materials and Methods

### 2.1. Study Population

The current study was conducted within the frame of a type 2 diabetes project in the Nankou Community of Changping, Beijing, in China between March 2014 and January 2015. A questionnaire of essential demographic information, including age, gender, previous medical history, and medication history, was assigned among 599 subjects who all signed written informed consent voluntarily.

Exclusion criteria include the following: (1) use of antidiabetic medications in the past three months with known diabetes; (2) use of lipid-lowering drugs or steroids in the past three months; (3) positive detection of antibodies related to type 1 diabetes including insulin autoantibodies (IAA), islet-cell antibodies (ICA), islet antigen-2 antibodies (IA2), and glutamic acid decarboxylase autoantibodies (GAD-Ab); (4) complication with cardiovascular and cerebrovascular diseases or chronic kidney diseases; and (5) refusal to the telomere length or mitochondrial copy number test. Eventually, a total of 450 subjects were included in this study. The clinical trial was approved by the ethics committee of Peking Union Medical College Hospital (ZS-1274).

### 2.2. Clinical Measurement

All subjects received a physical examination, including measurements of waist circumference (WC), hip circumference (HC), height, and weight (wearing lightweight clothes without shoes), and blood pressure was collected. Body mass index (BMI) was calculated as weight/(height × height) (kg/m^2^). Waist circumference (the level of the midpoint line between the iliac crest and the costal margin on both sides) and hip circumference (the level of the hip rotor) were measured twice by the same observer. The mean values were recorded. Blood pressure was measured twice using the same standard mercury sphygmomanometer at rest, and the mean value was calculated.

### 2.3. Biochemical Measurements

A 75 g oral glucose tolerance test (OGTT) was performed after overnight fasting. Blood samples were collected at 0, 30, 60, and 120 min following the OGTT. Plasma glucose was determined by the glucose oxidase assay. Lipid metabolism-related indices, including cholesterol (TC), triglyceride (TG), high-density lipoprotein (HDL-C), and low-density lipoprotein (LDL-C), were determined using an automated analyser (AU5800 automatic biochemistry analyser, Beckman Coulter). Chemiluminescent enzyme immunoassay (ADVIA Centaur XP, Siemens) was developed to quantify insulin and C peptide. HbA1c concentrations were assayed by high-performance liquid chromatography (D10 hemoglobin testing system, Bio-Rad; intra-assay coefficient of variation (CV) < 3%, interassay CV < 10%).

### 2.4. Assessment of Insulin Resistance (IR) and *β* Cell Function

The homeostatic model assessment of insulin resistance (HOMA-IR) was used to evaluate the degree of insulin resistance [4]: HOMA‐IR = fasting blood glucose (mmol/L) × fasting insulin (IU/mL)/22.5. Insulin secretion of the steady-state model (homeostasis model assessment of insulin secretion (HOMA-*β*)) was calculated as the evaluation of islet beta-cell function [4]: HOMA‐*β* = fasting insulin (IU/mL)/(fasting blood glucose (mmol/L) − 3.5).

### 2.5. Measurement of LTL

The determination of telomere length in peripheral blood has been described in detail previously [5]. QIAamp DNA blood mid kit (Qiagen, Hilden, Germany) was used to extract Genomic DNA in leukocytes. Purified DNA samples were diluted and quantified using a NanoDrop 1000 spectrophotometer (Thermo Fisher Scientific, Wilmington, DE, USA). The ratio of telomere repeat copy number to single-gene copy number (*T*/*S*) was determined by the novel monochrome multiplex quantitative PCR. The *T*/*S* ratio was used to indicate the telomere length [6]. The CV within the plate was 18%, and the interassay CV was 7%.

### 2.6. Measurement of Peripheral Blood mtDNAcn

The specific method of measurement of peripheral blood mtDNAcn has been described in detail. In brief, the nicotinamide adenine dinucleotide (NADH) dehydrogenase subunit 1 (ND1) genes were used as representatives of mitochondrial genes, and single-copy nuclear gene beta-actin served as the control gene. Quantitative RT-PCR was used to determine ND1 and *β*-actin reference genes quantitatively. The ratio of mtDNAcn to *β*-actin copy number represented the relative copy number of mtDNAcn. The ratio of each specimen was normalized to the alignment of the calibrated DNA samples. The intra-plate CV was 4.2% (1.6-9.8%), and the interassay CV was 4.6% (0.9-7.8%).

### 2.7. Measurement of Oxidative Stress and Inflammatory Indicators

Fasting blood samples were collected. Determinations of serum GR, SOD activity, TNF*α*, and IL-6 were done using the Elisa kit (Cloud-Clone Corp, Houston, USA) by the Beijing Institute of Biotechnology.

### 2.8. Statistical Analysis

All data were analysed using SPSS 22.0 (IBM Corp., Chicago, IL, USA). The normal distribution data were presented as the mean ± standard deviation, and parameters that were not normally distributed were expressed as the median (p25th, p75th). The data of the normal distribution were compared by Student's *t*-test, and the data not normally distributed were transformed. Nonparametric tests were used to analyse the data that could not be transformed. Correlation between data was assessed using Spearman's correlation analysis. Linear regression analyses were used to establish five sets of models to adjust for covariates that might affect the relationship between LTL and mtDNAcn. Model 1 was adjusted for age and gender. Model 2 was adjusted for antioxidant indices (GR, SOD activity) based on Model 1. Model 3 was adjusted for inflammatory indices (IL6, TNF*α*) based on Model 1. Model 4 made an adjustment of lipid metabolism indicators (TC, TG, HDL-C, and LDL-C) based on Model 1. The mediation analysis was performed by a three-step test model. In the first step, the independent variables were significantly correlated with the mediator variable. Next, the relationship between the independent variable and the dependent variable would be tested. The third step was to investigate the association between intermediate variables and the dependent variables controlling the independent variables. For the model with the insignificant three-step test, the Sobel test was used to verify the results again.

## 3. Results

### 3.1. Clinical and Demographic Characteristics in Groups with Different Glucose Tolerance Statuses

Based on the 1999 World Health Organization criteria, normal glucose tolerance (NGT, *n* = 193) was defined as fasting blood glucose (FPG) < 6.1 mmol/L and 2 h postprandial glucose (2hPG) < 7.8 mmol/L. Abnormal glucose metabolism (AGM) was classified as FPG ≥ 6.1 mmol/L or Glu120 ≥ 7.8 mmol/L. The general demographic characteristics of the two groups are shown in Table 1. Based on the Working Group for Obesity in China (WGOC) criteria, overweight and obesity were defined as 24 ≤ BMI < 28 and BMI ≥ 28 [7].

It was unexpected that the AGM group had a higher proportion than the NGT group (57.1% vs. 42.9%), which implicated the current situation of glucose metabolism in rural China was not optimistic. Compared with the NGT, the AGM was older (NGT vs. AGM: 49 ± 11.26 y vs. 55.21 ± 10.21 y, *p* ≤ 0.001) and had higher BMIs, larger WCs, and HCs (*p* ≤ 0.001). Over 450 subjects (41.5%, *n* = 187) were overweight, and 27.5% (*n* = 124) were obese. Systolic blood pressure was higher in the AGM (*p* = 0.01). The insulin resistance index HOMA-IR and the insulin secretion index HOMA-*β* of the AGM were higher than those of the NGT (*p* < 0.01). In terms of lipid metabolism, TC, TG, and LDL-C in the AGM were higher (*p* < 0.01), while HDL-c rendered no significant difference between the two groups (*p* = 0.05). No differences were found in oxidative stress and inflammatory factors (including GR, SOD activity, Il-6, and TNF*α*) between the NGT and the AGM. Among the aging indices, mtDNAcn was significantly decreased in the AGM (NGT vs. AGM: 108.88 ± 44.31 vs. 100.47 ± 41.04, *p* = 0.023), while LTLs in the AGM did not differ from those in the NGT (NGT vs. AGM: 28.72 ± 0.83 vs. 28.67 ± 0.851, *p* = 0.583).

### 3.2. MtDNAcn Was Positively Correlated with LTL in People with AGM and the Correlation Disappeared after Correcting Inflammatory Factors

**Figure 1:**
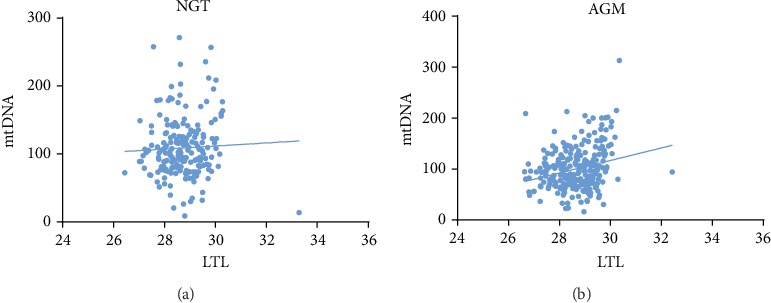
Correlation analysis between mtDNAcn and LTL in populations with different glucose tolerance statuses: (a) correlation analysis between mtDNAcn and LTL in the NGT (*r* = 0.071, *p* = 0.325) and (b) correlation analysis between mtDNAcn and LTL in the AGM (*r* = 0.214, *p* = 0.001).

Spearman's correlation analysis was used to explore the relationship between LTL and mtDNAcn in people with different blood glucose levels (Figure 1). In the NGT, no correlation was found between LTL and mtDNAcn (*r* = 0.071, *p* = 0.325). Among the subjects in the AGM, LTL was positively correlated with mtDNAcn (*r* = 0.214, *p* = 0.001). After adjusting for age and gender, the relationship between LTL and mtDNAcn did not significantly change (*p* ≤ 0.001) (Model 1 (M1)) (Table 2). Further analyses of the relationships of LTL and mtDNAcn were conducted separately after correcting oxidative stress, inflammatory indicators, and lipid metabolism-related indices based on adjusting for age and gender (Table 2). There was still a positive correlation between LTL and mtDNAcn after correcting antioxidant indices (GR, SOD activity) and lipid metabolism indices (*p* < 0.01). However, the correlation between LTL and mtDNAcn disappeared when we accounted for inflammatory indicators (IL-6, TNF*α*) (nonstandardized *β* = 6.237, *p* = 0.087).

### 3.3. The SOD Activity Was Negatively Correlated with FPG, and TNF*α* Was Positively Correlated with HbA1c and 2hPG in the AGM

The relationship between oxidative stress and glucose metabolism was analysed in AGM (Table 3). SOD activity value was negatively correlated with FPG (*r* = ‐0.161, *p* = 0.016), and SOD activity was not related to 2hPG, HbA1c, HOMA-IR, HOMA-*β*. No correlation was observed between GR and the indicators of glucose metabolism above.

In investigating the relationship between inflammatory and glucose metabolism indices in AGM (Table 3), TNF*α* was positively correlated with 2hPG (*r* = 0.247, *p* ≤ 0.001) and HbA1c (*r* = 0.16, *p* = 0.016), and TNF*α* was not correlated with FPG, HOMA-IR, and HOMA-*β*. IL-6 was not observed to be associated with indices of glucose metabolism above.

### 3.4. Telomere Shortening Was Associated with Increased HbA1c, Inflammatory Markers, and SOD Activity

**Figure 2:**
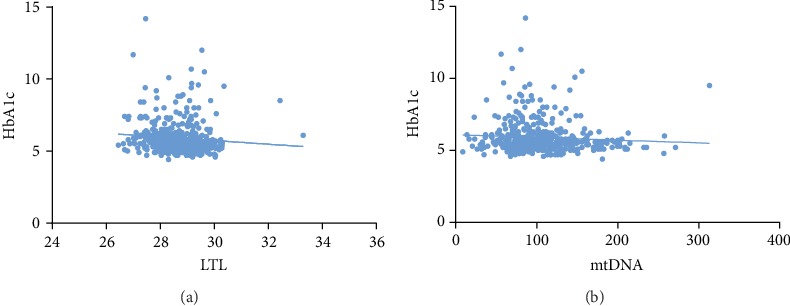
Correlation analysis between HbA1c and LTL and mtDNAcn: (a) correlation analysis between HbA1c and LTL (*r* = ‐0.174, *p* ≤ 0.001) and (b) correlation analysis between HbA1c and mtDNAcn (*r* = ‐0.085, *p* = 0.072).

LTL was negatively correlated with HbA1c (*r* = ‐0.174, *p* ≤ 0.001) (Figure 2(a)). Further analysis found that LTL was significantly negatively correlated with SOD activity (*r* = ‐0.236, *p* ≤ 0.001), IL-6 (*r* = ‐0.133, *p* = 0.008), TNF*α* (*r* = ‐0.477, *p* ≤ 0.001) (Table 4). There was no correlation between LTL and GR (*r* = ‐0.064, *p* = 0.205).

### 3.5. Decreased mtDNAcn Was Associated with Elevated SOD Activity, IL-6, and TNF*α* Levels

There was no statistical relationship between mtDNAcn and HbA1c (*r* = ‐0.085, *p* = 0.072) (Figure 2(b)). Decreased mtDNAcn was significantly associated with an elevated level of SOD activity (*r* = ‐0.236, *p* ≤ 0.001), IL-6 (*r* = ‐0.133, *p* = 0.008), and TNF*α* (*r* = ‐0.219, *p* = 0.001) (Table 4). MtDNAcn was not statistically correlated with GR (*r* = 0.040, *p* = 0.548).

### 3.6. TNF*α* Played a Mediating Role in Telomere-Mitochondria Interactome when Glucose Metabolism Is Abnormal

**Figure 3:**
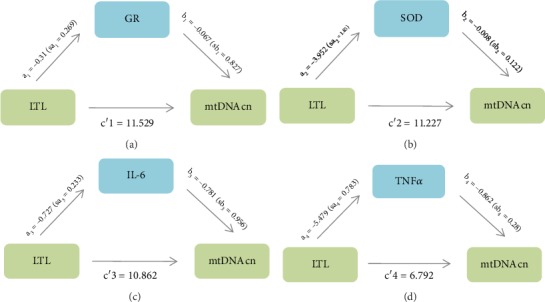
Mediational analysis of antioxidants and inflammatory indicators on the relationship between LTL and mtDNAcn after adjusting for age and gender. (a) a1: the effect of LTL on GR (*p* = 0.251); b1: the effect of GR on mtDNAcn (*p* = 0.925); a1 × b1: mediating effect of GR on mtDNAcn; c′1: the direct effect of LTL on mtDNAcn after the correction of GR (*p* = 0.001). (b) a2: the effect of LTL on SOD activity (*p* = 0.032); b2: the effect of SOD activity on mtDNAcn (*p* = 0.472); a2 × b2: mediating effect of SOD activity on mtDNAcn; c′2: the direct effect of LTL on mtDNAcn after the correction of SOD activity (*p* = 0.001). (c) a3: the effect of LTL on IL-6 (*p* = 0.002); b3: the effect of IL-6 on mtDNAcn (*p* = 0.415); a3 × b3: mediating effect of IL-6 on mtDNAcn; c′3: the direct effect of LTL on mtDNAcn after the correction of IL-6 (*p* = 0.001). (d) a4: the effect of LTL on TNF*α* (*p* ≤ 0.001); b4: the effect of TNF*α* on mtDNAcn (*p* = 0.002); a4 × b4: mediating effect of TNF*α* on mtDNAcn; c′4: the direct effect of LTL on mtDNAcn after the correction of TNF*α* (*p* = 0.058).

Through mediation analysis, it was found that TNF*α* played a full mediating effect in the positive correlation between LTL and mtDNAcn (c′ was not significant, *p* = 0.058). However, GR, SOD viability, IL-6 had no mediating effect on LTL and mtDNAcn (Figure 3).

## 4. Discussion

This study investigated the association between oxidative stress and inflammatory markers, LTL, and mtDNAcn in groups with different glucose tolerance status in a rural community of Beijing. Despite a higher prevalence of prediabetes, the diagnosis and treatment of hyperglycemia patients in rural China lag far behind to that in urban areas.

To minimize the effect of the medication, diabetic patients who had recently taken antidiabetic drugs, as well as the individuals who received lipid-lowering agents or steroids recently, were excluded. The present study found a higher prevalence of AGM, which was 57.1% in this rural community, than that (50.9%) in the previous report of Chinese rural areas [8]. Additionally, the study population has a larger number of people with overweight and obesity compared with the population studied in the northeast rural community of China in 2012, where the prevalence of overweight and obesity was 31.6% and 14.6% [9]. These data alerted us the prevention and risk factor management of aging-related metabolic disease in rural northern China is urgent. This real-world study revealed that LTL was positively correlated with mtDNAcn only in the AGM, and the correlation disappeared after the adjustment of the inflammatory indices. There was an inverse correlation between SOD activity and fasting blood glucose in the AGM, while TNF*α* was positively correlated with HbA1c level and 2hPG. At the same time, this study also found that LTL was negatively correlated with SOD activity, TNF*α*, and IL-6. The decreased mtDNAcn was found in those with higher SOD activity, TNF*α*, and IL-6 levels. The mediation analysis revealed that TNF*α* acted as a mediator between LTL and mtDNAcn.

Previous studies have confirmed a positive correlation between mtDNAcn and LTL in the elderly [10]. LTL and mtDNAcn are both hallmarks of aging, which might accelerate the progression of diabetes. There is abundant evidence that the abrasion of telomere could activate the tumor suppressor gene p53 and inhibit the expression of PGC1a, leading to the dysfunction of mitochondrial synthesis [11]. On the other hand, excessive acetyl-CoA in the mitochondria has been shown to increase the production of NADH resulting in an aggravation of telomere attrition. Our previous study has revealed that mtDNAcn is negatively correlated with plasma glucose levels measured at 30 and 120 min during 75 g OGTT in population with different glucose status [12]. The present study has indicated that LTL is positively correlated with mtDNAcn only in the AGM group, which indicated that the decrease in mtDNAcn might imply a decline of glucose-stimulate insulin secretion (GSIS) of *β*-cell [12]. The activation of telomere-mitochondrial interaction in the glucose intolerance population might induce deterioration of *β*-cell and insulin resistance. In other words, the vicious cycle between telomere shortening and mitochondria dysfunction might be formed by hyperglycemia. However, based upon the evidence of the cross-sectional characteristics, this study cannot provide a definitive causal link between the telomere-mitochondrial axis and hyperglycemia.

HbA1c, a critical biomarker that reflects long-term glycemic control, has its value in predicting the complications associated with aging among diabetic patients [13]. This study demonstrates that LTL is shortened with the elevation of HbA1c, indicating that telomere damage might be involved in poorly controlled hyperglycemia. However, no correlation has been found between mtDNAcn and HbA1c, which suggests that the interaction between telomere and mitochondria is not mediated by hyperglycemia itself. Inflammation and oxidative stress have been firmly established as central roles in the development of hyperglycemia, which is also a promoter of mitochondria disorder [14]. Although previous studies have proved that lipid metabolism and oxidative stress were closely related to inflammatory factors [15], this study suggests that neither lipid metabolism nor antioxidant indices affected the telomere-mitochondrial interaction. The present study shows that the positive correlation between LTL and mtDNAcn disappears after adjusting for inflammatory factors, including IL6 and TNF*α*, indicating that inflammatory factors are critical factors for the interaction between telomere and mitochondria.

Among several antioxidants and inflammatory markers, this study has revealed that only TNF*α* exerts a complete mediating effect on the telomere-mitochondrial interactome and confirmed that TNF*α* is negatively correlated with mtDNAcn on a higher level of HbA1c. However, how TNF*α* mediated the telomere-mitochondria axis in hyperglycemia remains unclear and has yet to be further explored. TNF*α* is a proinflammatory cytokine and can be downregulated by the activation of the SIRT1 (sirtuin1) gene. Research in the elderly has proved that the expression of the SIRT1 gene mediates a 40% positive correlation between LTL and mtDNAcn [16]. It can be hypothesized that the LTL shortening in hyperglycemia may increase the TNF*α* level by reducing SIRT1 activity, which leads to a reduction of mtDNAcn. The treatment aimed at lowering the TNF*α* level is expected to delay the progression of mitochondrion dysfunction caused by telomere attrition due to hyperglycemia. Meanwhile, the improvement of mitochondrion function can benefit the glucose uptake driven by the oxidative phosphorylation and slow down the cell senescence of individuals with hyperglycemia [17]. Given the limited evidence, further research is required to look into the specific mechanisms of TNF*α*-mediated telomere attrition and mitochondria dysfunction.

## 5. Conclusions

This is a cross-sectional study based on a noninterventional rural community in Beijing, China. Over half of adults in this rural population are found to have AGM by OGTT, including diabetes and prediabetes. It is also shown that mitochondrial dysfunction is closely related to telomere shortening, which is fully mediated by elevation of TNF*α* in AGM depending on hyperglycemia.

## Figures and Tables

**Table 1:** Clinical and demographic characteristics in groups with different glucose tolerance statuses.

Parameter	NGT (*n* = 193)	AGM (*n* = 257)	*p*
Age (years)	49 ± 11.26	55.21 ± 10.21	≤0.001^∗∗^
Sex, male : female^†^	62 : 131	97 : 160	0.120
BMI (kg/m^2^)	25.31 ± 3.55	26.64 ± 3.85	≤0.001^∗∗^
Waist circumference (cm)	84.94 ± 9.93	88.83 ± 9.88	≤0.001^∗∗^
Hip circumference (cm)	90.08 ± 9.93	88.83 ± 9.88	≤0.001^∗∗^
SBP (mmHg)	125.03 ± 19.00	130.36 ± 19.26	0.004^∗^
DBP (mmHg)	75.70 ± 9.43	76.4 ± 10.52	0.486
HbA1c%	5.3 (5.1, 5.6)	5.90 (5.5, 6.6)	≤0.001^∗∗^
HOMA-IR	2.22 (1.54, 3.07)	3.23 (2.16, 4.99)	≤0.001^∗∗^
HOMA-*β*	91.32 (67.99, 130.56)	65.09 (41.08, 98.29)	≤0.001^∗∗^
FPG (mmol/L)	5.46 (5.22, 5.72)	6.57 (6.13, 7.90)	≤0.001^∗∗^
2hPG (mmol/L)	6.03 (5.04, 6.89)	9.7 (7.83, 14.83)	≤0.001^∗∗^
TC (mmol/L)	5.30 ± 0.1	5.68 ± 0.1	≤0.001^∗∗^
TG (mmol/L)	1.20 (0.78, 1.66)	1.71 (1.13, 2.36)	≤0.001^∗∗^
HDL-C (mmol/L)	1.29 (1.11, 1.50)	1.25 (1.08, 1.44)	0.050
LDL-C (mmol/L)	2.71 ± 0.07	2.99 ± 0.07	≤0.001^∗∗^
GR (U/L)	7.20 ± 3.22	7.00 ± 3.33	0.538
SOD activity (U/mL)	60.69 ± 18.51	59.57 ± 18.57	0.46
IL-6 (pg/mL)	3.96 ± 3.33	3.98 ± 2.93	0.932
TNF*α* (fmol/mL)	23.96 ± 10.32	23.93 ± 10.67	0.977
LTL	28.72 ± 0.83	28.67 ± 0.851	0.583
mtDNAcn	108.88 ± 44.31	100.47 ± 41.04	0.023^∗^

^∗^ *p* < 0.05; ^∗∗^*p* < 0.01; ^†^chi-square test. NGT: normal glucose tolerance; AGM: abnormal glucose metabolism; BMI: body mass index; SBP: systolic blood pressure; DBP: diastolic blood pressure; HOMA-IR: homeostatic model assessment of insulin resistance; HOMA-*β*: homeostasis model assessment of insulin secretion; FPG: fasting plasma glucose; 2hPG: 2 h postprandial plasma glucose; TC: cholesterol; TG: triglyceride; HDL-C: high-density lipoprotein; LDL-C: low-density lipoprotein; GR: glutathione reductase; SOD: superoxide dismutase; IL-6: interleukin-6; TNF*α*: tumor necrosis factor *α*; LTL: telomere length; mtDNAcn: mitochondrial DNA copy number.

**Table 2:** Correlation analysis between LTL and mtDNAcn in a correction model of abnormal glucose metabolism.

Correction model	Unstandardized coefficients (*β*)	Standardized coefficients (*β*)	*R* ^2^	*p*
M1	11.884	0.245	0.073	≤0.001^∗∗^
M2	11.232	0.224	0.074	0.001^∗∗^
M3	6.237	0.125	0.112	0.087
M4	11.813	0.242	0.106	≤0.001^∗∗^

Model 1 (M1): adjusted for age and gender. Model 2 (M2): adjusted for antioxidants (GR and SOD activity) based on Model 1. Model 3 (M3): adjusted for inflammatory (IL-6 and TNF*α*) based on Model 1. Model 4 (M4): adjusted for lipid metabolism indicators (TC, TG, HDL-C, LDL-C) based on Model 1. ^∗^*p* < 0.05; ^∗∗^*p* < 0.01.

**Table 3:** Correlation analysis of antioxidants and inflammatory indicators with glucose metabolism indices in the AGM.

	HbA1c% *r*	*p*	FPG (mmol/L) *r*	*p*	2hPG (mmol/L) *r*	*p*	HOMA-IR *r*	*p*	HOMA-*β* *r*	*p*
GR (U/L)	-0.086	0.199	-0.052	0.436	-0.022	0.745	0.012	0.864	0.027	0.687
SOD activity (U/mL)	-0.037	0.587	-0.161	0.016^∗^	-0.13	0.052	-0.105	0.12	0.055	0.416
IL-6 (pg/mL)	-0.012	0.858	-0.09	0.179	-0.03	0.66	0.014	0.831	0.098	0.150
TNF*α* (fmol/mL)	0.247	≤0.001^∗∗^	-0.059	0.379	0.16	0.017^∗^	0.036	0.595	0.045	0.510

^∗^ *p* < 0.05; ^∗∗^*p* < 0.01. FPG: fasting plasma glucose; 2hPG: 2 h postprandial plasma glucose; HOMA-IR: homeostatic model assessment of insulin resistance; HOMA-*β*: homeostasis model assessment of insulin secretion; GR: glutathione reductase; SOD: superoxide dismutase; IL-6: interleukin-6; TNF*α*: tumor necrosis factor *α*.

**Table 4:** Correlation analysis of antioxidants and inflammatory indicators with LTL and mtDNAcn.

	LTL *r*	*p*	mtDNAcn *r*	*p*
GR (U/L)	-0.064	0.205	0.011	0.833
SOD activity (U/mL)	-0.229	≤0.001^∗∗^	-0.139	0.006^∗∗^
IL-6 (pg/mL)	-0.133	0.008^∗∗^	-0.144	0.004^∗∗^
TNF*α* (fmol/mL)	-0.477	≤0.001^∗∗^	-0.236	≤0.001^∗∗^

^∗^ *p* < 0.05; ^∗∗^*p* < 0.01. GR: glutathione reductase; SOD: superoxide dismutase; IL-6: interleukin-6; TNF*α*: tumor necrosis factor *α*; LTL: telomere length; mtDNAcn: mitochondrial DNA copy number.

## Data Availability

338 The datasets analysed in this manuscript are not publicly available. Requests to access the datasets should be directed to the datasets supporting the conclusions of this manuscript arebavailable from the corresponding author (pingfan6779@163.com or liyuxiu@medmail.com.cn) on reasonable request.
